# Statistical Report on the Sickness and Mortality in the Army of the United States

**Published:** 1857-07

**Authors:** 


ART. V.—*Statistical Report on the Sickness and Mortality in the Army of the United States. Compiled from the Records of the Surgeon-General's Office; embracing a period of sixteen years, from* 1839 *to* 1855. Prepared under the direction of Brevet Brigadier General THOMAS LAWSON; Surgeon-General U. S. Army. By RICHARD H. COOLIDGE, M.D., Assistant Surgeon U. S. Army.

LORD BACON observed that the great want of medical science was a closer "inquisition" into the causes of organic decay, and of the brevity or length of life; an inquiry needing to be made complete in all its parts, and confirmed by numerical calculations. If he estimated at its lowest point the acquirements of the profession, in his time, he has been more than followed, in this, by a bold usurper of many of his laurels,—M. Comte; who, after an allusion to "the profound imperfection of the existing education" of medical men, dismisses their scientific pretensions, thus: "If we would not confide the study of astronomy to navigators, we must not leave biology to the leisure of physicians."

Yet, if the study of the winds and the waves cannot be pursued without the aid of those who navigate the deep,—so pathology, at least, if not physiology, must depend essentially upon practical physicians for those facts which constitute its structure, and upon which its principles are to be founded.

It is to be regretted, that a much more systematic habit of accumulation of such facts does not generally prevail; since, bare and inert as each series may appear by itself, it needs but aggregation, with the electric spark of thought, to constitute of them an organization, endowed with all the vitality of science.

The industry of the army medical staff, during sixteen years, has brought together much of great importance, as to the medical topography and sanitary condition of the United States. A quarto volume, of seven hundred pages, having a considerable proportion of tabular matter, forms its representative embodiment.

We do not suppose that very many will read through this large volume; yet, having done so, we may pronounce, that it is not only worthy of perusal, with the woorali (or *woorara*), life may be restored, the poison being expelled with some of the secretions. Prof. Cl. Bernard has found that the woorali destroys neither the reflex power nor the properties of sensitive nerves, but only the power of motor nerves, although it leaves muscular irritability entire. As to the deduction that the vital property of muscles is proved to be independent of nervous action, by the fact that the woorali destroys the motor nervous power without altering muscular irritability, Prof. Bernard had drawn it long before Dr. Pavy, and from the same experiment.

but of careful study. It contains material for a climatology of the United States; gives the actual history, from observation, of all the principal diseases, sporadic, endemic, and epidemic, of the country; shows the local circumstances, and meteorological conditions, which have coincided with epochs of disease; and adds to all this, much information in regard to the natural history of the continent, including the ethnology of its human tribes. It would afford data for a hundred theories; but, better than this, it will add to that pile of slowly aggregating knowledge, which, like the coral islands, unseen for centuries, will at last reach the air, and become almost at once a world of life and fertility. The value and interest of this volume is, no doubt, much increased by the care and labor with which its editor, Dr. Coolidge, has compiled it; and by the fact that it has been delayed, until an amply sufficient amount of material has been collected.

The plan of the work is as follows: The territory of the United States is separated into three great divisions, the Northern, Middle, and Southern; beside which are enumerated, distinctly, Florida, Texas, New Mexico, California, and Oregon and Washington Territories. Most of these are again subdivided; as, the Northern Division, into the *North Atlantic* Region, including the coast of New England, and New York harbor; and the *North Interior*, including the posts east of, upon, and westward of the Great Lakes. The Middle Division is subdivided into the *Middle Atlantic*, and the *Middle Interior*, East and West; the Southern, into the *South Atlantic*, and *South Interior*. Other similar distributions are made of the regions in Florida, Texas, &c.

The Surgeon-General having issued, in 1852, a circular of inquiry to all the medical officers on duty, a reply was sent by each, upon the topography, climate, diseases, &c., of his locality. These reports are, many of them, elaborate, and very interesting. After those of each region, Assistant Surgeon Coolidge has inserted an abstract, prepared from the original reports, of the number of cases of sickness and death occurring in each section. At the end of the reports from each division, full abstracts of disease and mortality are inserted, in complete tables. Consolidated abstracts terminate this series; after which, there is an appendix, containing matter of great importance and interest.

A few pages will hardly suffice to convey an idea, by extracts or summaries, of the information contained in these reports. We propose, in this review, to give only such desultory notice as our limits allow, of those statements which seem to have the greatest *practical* value.

In the report of Assistant Surgeon Eaton (p. 16), an account is rendered of the medical topography of Fort Hamilton, in New York harbor. Every one is familiar with the fact that yellow fever occurred at that post and in its vicinity, last summer, and was ascribed to the contiguity of the shipping.

It is of importance, then, to find, as we do in this report, that Fort Hamilton is situated in an *insalubrious* locality; where those noxious vapors, proceeding from organic decomposition, exist, which some have supposed not to have had any influence in this instance.

Passing abruptly to another topic,—at Fort Kent, in Maine, on the St. John's River, an opportunity occurred of comparing the date of puberty, in descendants of different races, in the same climate. The mean temperature for the year at Fort Kent, is about 36°; it being in latitude 47° 15″ N.

Among the French inhabitants, of Acadian descent, "the average date of the first appearance of the menses, in 38 cases, was 13·5 years; in one case commencing at 11, in the most protracted at 19 years. To compare with these," says Assistant Surgeon Witherspoon, "I have procured the age at which the only eight Americans girls who have been raised on the river, first menstruated. The average date was 15·12 years, the youngest at 14, the eldest at 16. As the Americans have a decided superiority in manner of living, use better and more stimulating food, wear warmer and more comfortable clothing, all of which causes would tend to accelerate the period of puberty, it would seem that race as well as climate, has great influence in the matter."

We are struck with the comparative absence of serious disease of the respiratory organs, and especially of phthisis, at the posts in the North Interior region. This is noticed at Fort Kent, the most northerly of all in the United States; also at Forts Fairfield, Maine; Winnebago, Wisconsin; Dodge and Atkinson, Iowa; and Laramie, Nebraska. In New Mexico, the same thing has been observed; the difference in latitude being there compensated for by the elevation; which is, at all the posts, from 4000 to 8000 feet above the level of the ocean. These facts are alluded to by Assistant Surgeon Coolidge at the close of the volume, as the basis of some deductions which we will notice in their place.

At Madison Barracks, on Sackett's Harbor, eight miles from Lake Ontario, Assistant Surgeon Henderson has recorded the coincidence of a decided increase in the amount of malarious fever, with the *falling of the lakes;* a coincidence which, notwithstanding the opinion of the reporter, we cannot but think significant of partial causation.

Assistant Surgeon C. E. Isaacs gives an interesting account of a singular epidemic of *peritonitis,* which occurred at Copper Harbor, on Lake Superior, in the winter of 1844. It was fatal in but one case. At La Pointe, 180 miles farther west, a similar epidemic destroyed thirty of the inhabitants, in the winter of 1840.

Much information in regard to the habits and sanitary condition of the

Indian tribes, is afforded in the reports of Dr. David Day, Assistant Surgeons Keeney, Head, Hasson, Barnes, Moses, and others.

Whatever may have been the state of these tribes, before the white man overran the country, it would appear that civilization, with the evils which attend it as its shadow, is incompatible with their continued existence. Their mortality is greater than that of any other race or class on this continent, not excepting (as statistics show) the negroes, in any locality. Syphilis, small-pox, cholera, measles, and even remittent and intermittent fevers, aggravated by intemperance, superstition, and ignorance, are destroying them by thousands every year. Of the Osages alone, in 1845, there were six thousand; in 1850, five thousand; in 1852, but thirty-five hundred were left.

In the most extended paper in the volume, Surgeon John B. Porter gives the history of the climatology and diseases of Fort Moultrie, and Sullivan's Island; the interest of which is chiefly to be seen in connection with yellow fever. By an elaborate investigation of every epidemic, and nearly every case, which has occurred in those localities, he has established the *indigenous* origination of this fever, and the absence of any evidence of contagion. It is but fair to state, that Assistant Surgeons Hammond and McParlin, hold different views, based upon the observation of epidemics at Barrancas Barracks, and Pascagoula. The treatment of yellow fever, which Surgeon Porter prefers, consists chiefly in the administration of quinine and calomel, in what, at the North, would be considered pretty free doses. This is also the favorite plan of medication in the same disease with all but one or two of the other authors of the "statistics." We need not remark that experience, since the date of most of these reports, has shaken the confidence of many of the advocates of the "abortive" treatment, and thrown a preference upon the "palliative" plan, on the idea that we possess no specific or antidotal remedy for "typhus icterodes."

Dr. Coolidge, while on duty at Fort Gibson, near the Arkansas, furnished an account of *typhoid pneumonia*, which, as this disease is by some Northern physicians believed to be a "myth," we may quote. It is corroborated by the frequent mention of the same disease, as the "winter fever" of miasmatic regions, in other reports in this work:

"It usually attacks persons enfeebled by climate and malarial influences, who live in open houses, are poorly clothed, and, above all, intemperate. No case has occurred, to my knowledge, among the regular toops. The disease is sometimes rapidly fatal, being ushered in with a chill, during which the brain or lungs, or both, become fatally congested; the patient never rallying. In less severe cases, there is usually a chill, followed by fever, complicated with pneumonia, which is not unfrequently double."

"The attendant fever appears to be continued, and not to differ from the symptomatic fever accompanying pneumonitis at the North; but if closely watched, slight remissions may be observed, even in the severest cases. There appears to be an 'essential fever,' with local inflammation. In such cases, my observation tells me that you may bleed, cup, give mercurial cathartics, and use the tartrate of antimony freely, and still the disease will gain ground. I have closely watched several cases, and notwithstanding the employment of remedies which in ordinary pneumonia would have been successful, I have found the disease steadily advancing, both in extent and degree. In this condition, with extensive inflammation of both lungs, I have given the sulphate of quinine in ten and twenty grain doses, with the happy effect of *removing almost entirely the attendant fever* in less than twenty-four hours, and checking, or at least enabling remedies before inefficacious to check, the progress of the disease."

Another season, Dr. Porter writes thus on the same topic:

"The treatment adopted was moderate bleeding, cupping, and external irritants; a mercurial cathartic, followed by the nitrous powder of the United States Dispensatory, frequently repeated, and quinine given in sufficient doses to check the fever, which it always did.

"The disease had a typhoid tendency, which prevented large bleeding, and rendered the use of serpentaria, senega, and wine necessary in the latter stages.

"Sixteen cases were treated during this quarter, two of which were fatal; of these, one was apoplectic, when first seen; the insensibility was never removed, the patient dying in sixty hours. The other was complicated with meningitis, and was under treatment only thirty-six hours."

During the same winter (1846–7), a similar description of "congestive pneumonia" was given by Assistant Surgeon J. H. Bailey, as it occurred at Fort Smith, in the State of Arkansas; but his views of treatment were different. He states that

"Large doses of quinine were of no utility; small doses seemed, in some instances, beneficial in bolstering up the patient. Emetics, especially of sulphate of zinc and copper, often gave temporary relief. Calomel was always decidedly beneficial, and opium of more use than all other remedies together."

Looking over the accounts of *cholera*, we find that the earliest occurrence of it, at any post in the United States, was December 21st, 1848, at Lavacca, Texas; but it was in New Orleans also, in the same month.

In 1849, it was at Fort Columbus, in New York harbor, but not on the coast of New England; it prevailed during the same year in all the other regions within the limits of the United States, and in Texas. California, New Mexico, Oregon, and Washington Territories, were not visited. In 1850, 1851, and 1852, it was reported in the North Interior region west of the great Lakes, in the Middle Interior West, and in the South Interior; and in 1852, a description is also given of its ravages among the troops at Panama, *en route* for California. In 1854, it was again in New York harbor, as well as West of the great Lakes, and in the South Interior region. The greatest mortality, in proportion to cases, viz., 1 in 2·8 and 1 in 2·1, was at Jefferson Barracks and St. Louis Arsenal, places of noted insalubrity, and at Leavenworth, Kansas, where it was said to have been "brought by the troops from St. Louis." The lowest mortality (1 death in 14 cases) occurred in the Middle Interior region West of the great Lakes.* The treatment in vogue at nearly all the posts in the army, was the use of calomel and opium, as the main remedies, aided by diffusible stimulants and antispasmodics, with external warmth and stimulation. Some of the medical officers gave from 100 to 300 grains of calomel in twenty-four hours, "with success." No means are afforded to *compare,* satisfactorily, the results of different modes of treatment.

We have no room for farther quotation in regard to cholera, merely remarking that only one of the reporters (Surgeon De Camp) avows a belief in the contagion of this disease. At Fort Laramie, Nebraska, and in its vicinity, opportunity was given to remark upon the existence, in June, 1850, of an epidemic *on the road* which was traversed by numbers of emigrants, on their way to California and Oregon, extending to a distance of 470 miles, and then abruptly ceasing at the crossing of the Platte River. "The cholera was confined to the road, and among the emigrants. Many Indians remained on the road through curiosity, or for the purpose of begging; they paid a terrible penalty. Other bands of Indians, wiser than the above, left the road, and escaped." (p. 80.)

An epidemic of *influenza,* of considerable violence, swept over the whole country in 1843; it was noticed at nearly all the posts. Evidence cannot be obtained, from the recent medical history of the army, in favor of the hypothesis, that a connection, or even a coincidence exists between the periods of this epidemic and those of cholera.

Much information is furnished in these reports in regard to the occurrence and treatment of *scurvy.* The remedies which seem to have been most promptly successful, were the *maguey* (agave Americana), the prickly pear (cactus opuntia), and a diet of wild onions.

We have already remarked upon the comparative exemption from tuberculous disease of so many of the northern posts. The tabular statement given by Dr. Coolidge (p. 496), makes this still more evident; as, "with the exception of West Point, the lowest ratio of cases of consumption is in New Mexico, being 1·3 per 1000; and the highest in the South Atlantic regions, where it is 9·2 per 1000." Dr. Coolidge concludes, most rationally, that *dryness* and *equability* are vastly more important to the safety

* In the North Interior region West of the great Lakes, the mortality was but 1 in 10 cases.

of those threatened by or suffering from consumption, than temperature; and that a uniformly *low* temperature is, for them, preferable to one uniformly *high*, especially if the latter be accompanied by humidity.

We might occupy many pages, with interest to ourselves, if not to the reader, by discussing the data given in this book, in regard to the "nosogeography" of the indigenous *fevers* of our country. Intermittent and remittent fevers have disappeared (except when imported) from the New England States. They occur, however, annually, with different grades of violence, in all the other divisions of the United States, with the exception of Oregon and Washington Territories, and, perhaps, New Mexico. Most generally, the coincidence with slow streams and broad marshes, which is proverbial, exists in those places of prevalence described by the contributors to the army statistics; but exceptions occur. For instance, at Forts Scott, Atkinson, and Dodge, near the western borders of Missouri and Iowa, intermittent prevails yearly; although the air is *remarkable for dryness;* the only fact to be connected with miasmatic development being the luxuriant vegetation of the open prairie. Again, at Fort Riley, in Kansas, an example was given, in 1853, of the cutting down of immense quantities of timber early in the summer, leaving much vegetable matter to decay in the bottom, without producing any fever. At Monterey, California, where the vicinity of large lagoons, &c., might lead us to expect their occurrence, miasmatic diseases do not originate; while at Camp Far West, in a similar latitude and climate, but *twenty-five miles* from any marsh whatever, they abound. The pernicious or "congestive" type of fever is indigenous, as is well known, only in the southern zone of the United States. It was in regard to the treatment of this malignant endemic, that Surgeon-General Lawson instituted an inquiry, in 1843, which has produced some of the most interesting papers in this volume; upon the subject of the administration of *quinine in large doses.* Surgeon-General Lawson claims for the medical staff of the army the credit of having *established* this practice in Southern fevers.

A careful perusal of these papers (relating chiefly to the history of the fevers of Florida) has led us to conclude, that they give no ground for changing the practice common in the North, in regard to *ordinary* autumnal fevers; but that, in those of the so-called congestive or pernicious type, occurring *especially in persons who have been enfeebled by long exposure to miasmata, the large doses are not only safe, but indispensable.*

The *tables* of this volume, alone, might present a wide and fertile field of study. The amount of labor spent in their preparation and compilation must have been immense. Besides the special statistics of each separate region, consolidated abstracts (as we have already remarked) are given, of the whole number of cases of diseases, and the resulting mortality, for the sixteen years. Thus, for example, we notice that in the entire army, during that period, 1628 cases of cholera have occurred, with 598 deaths (1 in 2·74); of yellow fever, 723 cases, and 209 deaths (1 in 3·45); of remittent fever, 10,299 cases, and 210 deaths; of "common continued fever," 2207 cases, and 23 deaths; of typhus, 557 cases, and 118 deaths; &c. &c.

Exclusive of cholera, the annual proportion of deaths (from disease) to the numerical strength of the army, is 1 in 38·04, or 2·58 per cent.; but, as Dr. Coolidge remarks, this is really too high, as the excess of deaths in 1849 was probably in part owing to diseases contracted in Mexico during the war.

Of the different regions, the lowest mortality (4 per 1000 of mean strength) is seen to be at West Point; the next (9 per 1000) on the Coast of New England. The highest, is on the Gulf Coast of Florida, and at Jefferson Barracks and St. Louis Arsenal, Missouri; the former being 52·8, and the latter 47 deaths per 1000 of mean strength. By reference to another table, we find that the preponderance on the Gulf Coast of Florida, especially, is due to *fevers;* of which that region presents the largest proportion. The *fatality* of fevers, however, would seem, according to the reports, to be greatest in Northern California; viz., 1 death in 14 cases; while, on the Western Frontier of Texas, it is but 1 death in 961 cases; a difference so great, as to suggest the possibility of some error.

The Meteorological Tables are, also, of great interest. We can select but one or two of the most striking points. On examination, we find that the *coldest* posts in the United States are Forts Kent and Fairfield, Maine, and Fort Gaines in Minnesota; the former averaging for the year, respectively, 37·04° and 38·11°; the latter, 39·30°. The *hottest* stations are, at Key West and Fort Myers, South Florida, and Fort Yuma, California. At Key West, the average for the year is 76·43°.

The greatest average quantity of rain for the year, fell at Fort Pike, Louisiana, being 71·92 inches; the least, at the posts in New Mexico (from 6 to 12 inches), and at Fort Yuma, California, where the mean of two years gave but 3·24 inches. We are struck with the difference, in some instances, in this respect, between localities not very distant from each other; as, in Florida, the amount for the year at St. Augustine, was but 31·80 inches; at Fort Pierce, in the same State, 62·98. So, also, in Oregon, at Fort Orford, the most western post on the continent, there fell 68·52 inches in the year; at Fort Dallas, on the Columbia River, only 14·32 inches.

In a note appended at the close of the volume, by Surgeon-General Lawson, it is stated that his main objects in causing its preparation were, to encourage the medical officers in the habit of observing and recording facts in medicine and the collateral sciences, and to bring them into more direct communion with the medical profession in civil life, by adding their contributions to the general stores of knowledge. Both of these ends have, we are sure, been amply and satisfactorily attained; and the reporters and compiler have, by the presentation of this work, involved the civil members of the profession in a debt of obligation, which, with all their apparent advantages, they may long find it difficult to repay.

